# Breath Analysis as a Potential and Non-Invasive Frontier in Disease Diagnosis: An Overview

**DOI:** 10.3390/metabo5010003

**Published:** 2015-01-09

**Authors:** Jorge Pereira, Priscilla Porto-Figueira, Carina Cavaco, Khushman Taunk, Srikanth Rapole, Rahul Dhakne, Hampapathalu Nagarajaram, José S. Câmara

**Affiliations:** 1 CQM—Centro de Química da Madeira, Universidade da Madeira, Campus Universitário da Penteada, Funchal 9000-390, Portugal; E-Mails: priscillaportofigueira@gmail.com (P.P.-F.); carina-cavaco@hotmail.com (C.C.); jsc@uma.pt (J.S.C.); 2 Proteomics Lab, National Centre for Cell Science, Ganeshkhind, Pune 411007, India; E-Mails: khushmanlord@gmail.com (K.T.); rsrikanth@nccs.res.in (S.R.); 3 Laboratory of Computational Biology, Centre for DNA Fingerprinting & Diagnostics, Hyderabad, Andhra Pradesh 500 001, India; E-Mails: rahuldhakne@cdfd.org.in (R.D.); han@cdfd.org.in (H.N.); 4 Centro de Ciências Exatas e da Engenharia da Universidade da Madeira, Campus Universitário da Penteada, Funchal 9000-390, Portugal

**Keywords:** Exhaled Breath (EB) analysis, Disease diagnosis, volatile organic compounds (VOCs), volatile fingerprint, breath analysis based disease diagnosis (BADD)

## Abstract

Currently, a small number of diseases, particularly cardiovascular (CVDs), oncologic (ODs), neurodegenerative (NDDs), chronic respiratory diseases, as well as diabetes, form a severe burden to most of the countries worldwide. Hence, there is an urgent need for development of efficient diagnostic tools, particularly those enabling reliable detection of diseases, at their early stages, preferably using non-invasive approaches. Breath analysis is a non-invasive approach relying only on the characterisation of volatile composition of the exhaled breath (EB) that in turn reflects the volatile composition of the bloodstream and airways and therefore the status and condition of the whole organism metabolism. Advanced sampling procedures (solid-phase and needle traps microextraction) coupled with modern analytical technologies (proton transfer reaction mass spectrometry, selected ion flow tube mass spectrometry, ion mobility spectrometry, e-noses, *etc.*) allow the characterisation of EB composition to an unprecedented level. However, a key challenge in EB analysis is the proper statistical analysis and interpretation of the large and heterogeneous datasets obtained from EB research. There is no standard statistical framework/protocol yet available in literature that can be used for EB data analysis towards discovery of biomarkers for use in a typical clinical setup. Nevertheless, EB analysis has immense potential towards development of biomarkers for the early disease diagnosis of diseases.

## 1. Introduction

Non-communicable diseases, such as cardiovascular (CVDs), oncologic (ODs), neurodegenerative (NDDs), and chronic respiratory diseases, are the major causes of death in the developed countries [1]. They are also becoming highly prevalent in the developing countries due to changing lifestyles. It is estimated that by the year 2020, seven out of 10 deaths in developing countries and 80% of the global disease burden would be caused by non-communicable diseases [1]. For example, Diabetes is a major concern for the developing countries, as its incidence is projected to triplicate from 84 to 228 million cases during 2000–2030 period [2]. Taking together with the high incidence of infectious diseases, such as AIDS, malaria and different haemorrhagic viral infections, there is a double disease burden on developing countries [3]. There are two key contributing factors for this highly negative prognosis. They are: (a) Late diagnosis, usually performed using invasive and expensive procedures, when the diseases have already reached a life-threatening stage and (b) the critical lack of medical and laboratorial infrastructures [4]. Hence, there is an urgent need for development of new efficient diagnosis tools, particularly, those which reliably detect diseases at their early stages. Such technologies have to be cheap, reliable, rugged and portable, otherwise they may not be within the reach of most of the people living in the developing countries [3]. From this standpoint breath analysis based disease diagnosis (BADD) looks very promising and attractive. In this review, we focus on BADD discussing on the current trends and innovations from sampling procedure to the final data analysis and diagnosis opportunities. Regarding the EB volatile composition, we discuss the metabolic profiles of the most interesting volatiles and present a few selected examples of putative biomarkers. Finally, we will also discuss the data analysis processes followed in literature and their relevance in the light of the complexity of data generated from EB studies.

## 2. Exhaled Breath (EB) Analysis

Breath analysis is possibly one of the oldest forms of diagnosis. Its usage for disease diagnostics dates back to ancient Greeks where physicians used EB to diagnose different diseases [5,6]. Breath odours allow correct associations to certain diseases. For example, the sweet smell of diabetic ketoacidosis, the rancid odour of *C. difficile* stools [6], the fishy smell of breath associated to liver illness, the urine-like odour of kidney disease, the grapes flavour of *Pseudomonas* infections [5,7] or the sewer smell of the breath of patients with lung abscesses, caused by the proliferation of anaerobic bacteria [8,9,10,11]. The reason behind this capability is certainly the powerful human olfactory system that Bushdid *et al.* [12] recently demonstrated to be able to discriminate at least 1 trillion different olfactory stimuli. EB composition reflects the volatile composition of the bloodstream, and its composition can be correlated with the arterial concentration of the same analytes, although we have to consider that some volatile compounds are originated in the airways, not being present in the blood (as nitric oxide, for instance) [13]. Regardless of their origin, it would be very difficult to detect most of the volatiles directly from blood samples [14]. EB is mainly composed of nitrogen (N_2_), oxygen (O_2_), carbon dioxide (CO_2_), water vapour and inert gases. O_2_ and CO_2_ diffuse passively between blood and breath according to their concentration gradients across the alveolar-capillary junction, dragging together thousands of other very low abundant volatile organic compounds (VOCs), as long as they exhibit significant vapour pressures [15]. These VOCs, estimated in over 3000, account for less than 100 parts per million (ppm) of the total breath volume [13,16,17], although part of them, as acetone, isoprene and propanol, are more abundant, existing in the ppm to sub ppm range, while ketones, aldehydes and pentane, for instance, occur at even lower concentrations, at the parts per billion (ppb) to parts per trillion (ppt) levels [18,19,20,21]. It is precisely the combination of these VOCs that define the “smell” of EB. Many of them are systemic or endogenous, being produced in physiological processes, but the metabolic routes behind their production are known only for a very limited number of VOCs (reviewed in [13]). Other VOCs are exogenous and result from external contamination through the inhaled air or ingested foods or drinks. These are considered as pollution or background noise [22]. Unfortunately, this seems to be the case for most VOCs. Phillips *et al.* [10] analysed the EB from 50 normal individuals and processed the data obtained considering that only the VOCs with positive alveolar gradient (concentration higher in breath than in air) are more likely to be endogenous and not inhaled from external environment through the lung. They found that only half of the 340 VOCs identified fulfilled this condition. Moreover, they also reported a broad sample variation, because only 27 of these endogenous VOCs were present in all individuals [10]. This result is illustrative of the complexity of the EB samples and the existence of many contaminants originated by the ambient surroundings. In addition there are also other problems related with the subject or the analytical approach selected, as discussed by van de Kant *et al.* [23] for VOCs analysis in pulmonary diseases. Regarding the environmental influence, besides the contamination with ambient VOCs, the abundance of CO_2_ and water vapour in exhaled air, is particularly challenging as water condensation interferes with the quantification of low abundant VOCs, particularly alcohols and aldehydes [24]. The subject influence is also very pronounced because EB is highly dependent of the metabolism and therefore of the clinical characteristics of the subject, as age, gender, weight, diet, smoking habits, medication use, lifestyle and physical condition and existence of different diseases (liver impairment, diabetes, *etc.*) ([25,26]). Concerning the analytical aspects, there are again many bottlenecks in EB analysis: (a) lack of standardization of the experimental procedures, which would avoid broad variations in the results and (b) poor storage solutions or the deficient reliability of the available prototypes for EB real-time analysis. Nevertheless, breath analysis is having a large potential to offer an inexpensive, rapid and non-invasive diagnostic tool for several diseases [27,28,29,30,31]. Therefore, there is nowadays a consensual awareness throughout the scientific community that there are changes in the EB volatile composition that will allow us to obtain relevant information for diagnosis of several diseases, including CVD, NDD, OD, respiratory infections and diabetes. EB volatile composition has the potential to assess not only disease diagnosis, but also its severity, progression and response to treatment, although many improvements have to be done at the methodological level to achieve this goal.

### 2.1. EB Analysis Experimental Layout

**Figure 1:**
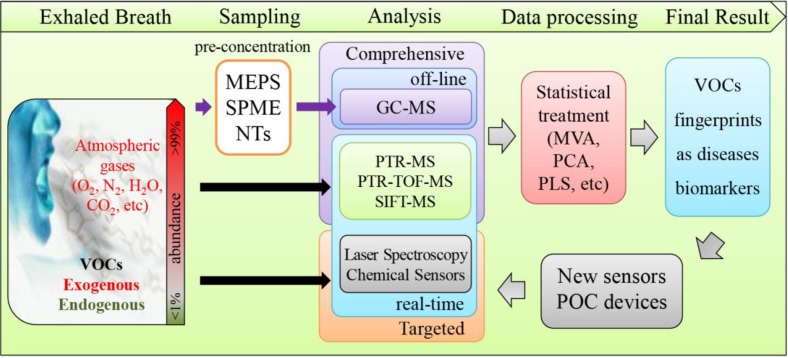
Generic layout for exhaled breath (EB) analysis. Abbreviations used: GC—gas chromatography, MEPS—microextraction by packed sorbent, POC—point of care, PTR-MS—proton transfer reaction with mass spectrometry, PTR-TOF-MS—proton transfer reaction with time-of-flight mass spectrometry, SIFT-MS—Selected ion flow tube mass spectrometry, NTDs—Needle Trap Devices, MVA—multivariated analysis, PCA—Principal Component Analysis, PLS—Partial least-square, VOCs—volatile organic compounds

EB analysis is technically challenging and could involve several experimental steps, depending on the methodology used. It is, therefore, prone to several experimental errors that will affect the quality of the results obtained if proper care is not taken. Moreover standard protocols/recommendations are not yet available albeit, many discussions about possible procedures in breath analysis. Nevertheless, as can be seen in Figure 1, EB analysis involves mainly three sequential steps: (a) Sampling and pre-concentration; (b) Analysis, processing of the data obtained and (c) Result output. Depending on the methodology selected, sampling and pre-concentration can be bypassed when EB is analysed in an online and real-time fashion. The statistical data processing that follows the sampling step can be particularly cumbersome due to data complexity in relation to sample size however. There is a full range of tools available to handle data complexity. However, there seems no consensus on selection and usage of tools to achieve discovery of volatile biomarkers that work with acceptable sensitivity and specificity for clinical applications.

#### 2.1.1. EB Sampling

**Figure 2:**
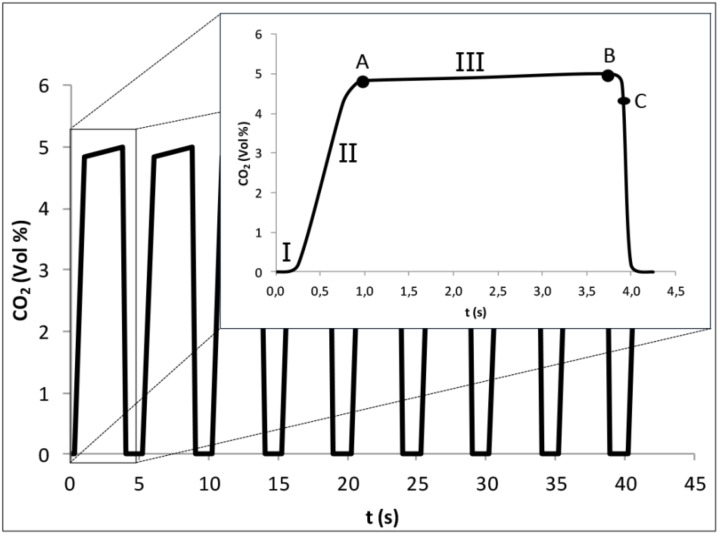
Exhaled CO2 monitoring by normal Capnograph: (**A**)—initial of alveolar sampling; (**B**)—final of alveolar sampling; (**C**)—final of exhalation. Differentiation phases of breathing: (**I**)-inspiratory phase; (**II**)-mixing phase; (**III**)-alveolar phase (adapted from [34,35]).

EB sampling is one of the most important steps in breath analysis and there are a number of parameters that researchers should pay attention, in order to avoid wrongful assumptions about the origin of the compounds identified. These parameters include the type and the number of breath collections, the portion of breath used, the EB storage and the interference of environmental VOCs from the collection room (reviewed in [24,32,33]). Breath collection can be achieved through a single breath or multiple breaths; both show associated advantages, which should be taken into account before choosing one of the two. For screening of potential compounds associated with a given disease and determination of a specific set of biomarkers, multiple breath analysis is required in order to achieve the best results, since it is more reproducible in terms of composition of the sample (reviewed in [24]). However, a single breath tends to be less time consuming and thus more acceptable for patients. EB can also be sampled in its full composition (total breath sample or mixed expiratory air) or, alternatively, only the alveolar air may be sampled. The first choice is more prone to contaminations, since the sampling control is very deficient. The patient just breathes for the sample collection device and therefore the risk of contamination with exogenous compounds from the oral cavity and dilutions in dead space are higher and may compromise the analysis (reviewed in [24]). These problems are reflected in the variation of the number of compounds and their concentration, jeopardizing the analytical reproducibility. In contrast, the alveolar air is richer in volatile blood-borne compounds [34]. Therefore the use of an alveolar air sampling approach, which can easily be achieved using a simple Capnograph to monitor the expiratory CO_2_ (Figure 2), is more accurate, assuring a more reliable sampling and consistent sample quality for within- and between-subject comparisons [34,35]. It would, therefore, be highly desirable that standardized procedures could be adopted for generic EB analysis as it was done for measurement of exhaled lower respiratory nitric oxide (NO) and nasal NO.

EB sampling can be performed directly or indirectly according to the type of analysis to be performed; we will see in more detail in the next section. In the off-line approach, EB has to be stored and concentrated before it is analysed. This procedure allows enrichment of the sample in the target analytes before its analysis [32]. EB storage can be made in the following ways [24,36]. The first approaches used Tedlar^®^ bags and canisters. The sampling bags are made from chemically inert materials as polyvinyl fluoride (PVF), perfluoroalkoxy polymer (PFA), polytetrafluoroethylene (PTFE) or polyvinylidene chloride (PVDC) [37]. These bags are impermeable to gas diffusion if covered with an aluminium foil [38] and their utilization is easy as they just need to be connected to a sample collection tube for EB collection. Tedlar^®^ bags are reusable provided they are extensively purged with nitrogen flows to avoid problems of contamination and background VOCs [27,32,36,37,39], such as some phenols and *N*, *N*-dimethylacetamide. These compounds can be released in relatively higher concentrations, thus contaminating the samples [40]. Despite these advantages Tedlar^®^ bags are vulnerable to punctures and some breath constituents, such as hexanal and isoprene cannot be stored for more than a few hours, thus limiting the utility of Tedlar^®^ bags for typical clinical studies [41,42]. It is possible that Tedlar^®^ bags can potentially give rise to biased results in which cases use of Tenax tubes is more appropriate [43,44]. Canisters are stainless steel recipients with an electropolished inner surface. This is necessary to reduce the adsorption of certain compounds and prevent their loss. Canisters are robust with long shelf-life [32,37], but their utilization is not suitable for sample collection (costs, space, storage, *etc.*) [37]. Another way to collect breath samples is using gas-tight syringes. A 50 mL syringe, for instance, is connected to a mouthpiece and patients breathe normally. Typically 10–20 mL of EB is drawn into the syringe and transferred to pre-evacuated glass vials (20 mL), where EB is stored before analysed. This procedure can be used both for mixed air as well as alveolar air [35,45]. The EB is saturated with water vapour and therefore can be condensed with cooling [46]. This exhaled breath condensate (EBC) is another form of sampling storage, but it is out of the scope of this review and extensive descriptions about it can be found elsewhere [47,48,49,50,51].

#### 2.1.2. Pre-Concentration

As already mentioned, the volatile composition of interest in EB represents less than 1% of its composition and can range from μmol L^−1^ to fmol L^−1^ [25,35]. Therefore, small interfering compounds could affect the analytical results. To minimize this effect, an intermediate step between sampling and analysis is sometimes necessary and advantageous to increase the concentration level of the target analytes over the possible interfering compounds. There are several enrichment techniques available, such as cryogenic trapping, usually used with the canister breath sampling, and adsorption in different thermal desorption tubes (TD-tubes), sorbent traps and coated fibres. The adsorption option requires a thermodesorption step, being usually followed by gas chromatography combined with mass spectrometry (GC/MS). A wide range of TD-tubes, also known as sorbent tubes, are available with different *strength* abilities to retain VOCs, working temperatures and hydrophobicity (reviewed in [52,53]). Therefore, applications of TD-tubes are also very broad and include, among others, environmental control [54], natural products characterisation [55], and breath research for disease diagnosis (as reported in [43,56,57,58]) (Table 1). Among the TD sorbents, Tenax tubes are quite popular for breath analysis as they allow a good performance in terms of pre-concentration and transport of breath samples before processing [43,56,57,58,59]). The multibed versions of TD-tubes, in which different sorbents with increasing strength are packed sequentially, are particularly suitable for breath analysis given the broad range in volatility of the VOCs present in EB samples [52]. Among the EB sample concentration methods, Solid-Phase Microextraction (SPME) is the most popular adsorption methodology [32,35,45,60,61]. This technique, particularly its headspace variant (HS-SPME), in which the extraction of the analytes belonging to solid or liquid sample is taken from the headspace, has gained tremendous importance with regard to the EB sampling. Based on the equilibrium partitioning of the analyte in the sample matrix and stationary phase, the HS-SPME extraction/concentration is more effective from the moment the analyte reaches the equilibrium concentration in the fibre, and its concentration in the extraction fibre remains constant [62,63,64]. The HS-SPME device is composed of a coated, silica fibre, with a thin layer (5–100 nm) of a suitable polymeric adsorbent. Many types of adsorbent are available in the market, for example, PDMS, CAR, DVB, combinations of these, and others. The selection of the fibre type and its thickness is usually done according to the polarity and molecular weight of the VOCs. In EB analysis, the CAR/PDMS stationary phases have shown great performance, however, the search for improved materials is still ongoing [62,63,64,65]. Additional factors affecting SPME performance, such as temperature and time of extraction, sample pH and ionic strength, are very important and must be optimized to obtain the best results. A more detailed overview about this experimental optimization can be found elsewhere (reviewed in [4,66,67,68]). An emerging and promising alternative that combines the EB sampling and pre-concentration steps in a single device is the Needle Trap Device (NTD) [69,70,71,72]. The NTD is a trap device composed of a needle containing a sorbent material packed inside. The sorbent constitution is variable and includes Carboxen (CAR), Divinylbenzene (DVB), Polydimethylsiloxane (PDMS), mixtures of these, and other sorbents (similar to the composition of SPME and TD-tubes). In the NTD, the sample can be actively drawn in and out by diffusion, gas-tight syringe or automated devices, such as vacuum pumps. The sensitivity that can be achieved using NTDs is comparable to SPME. Unlike SPME, NTD is an exhaustive methodology, allowing an increase in the concentration of several compounds by using more sample volume. The working principles behind NTDs are quite similar to the TD-tubes. However, their use is technically easier and straightforward since the steps, sampling, pre-concentration and sample injection (that can be made in a regular GC using thermal desorption and a lower internal diameter liner) occur sequentially and using a single device. Moreover, sample storage, prior to analysis, is also possible and has been shown to deliver reproducible results for several days or even weeks of storage, depending on the target analytes [41]. In summary, given the particularities and requirements of breath analysis, NTD seems to present substantial advantages over TD-tubes and SPME and its popularity will certainly grow exponentially in the next years. Similar to TD-tubes, the NTD multibed configurations are the most appropriate to retain the wide volatile composition of EB [41].

### 2.2. EB Analysis

Since Pauling *et al.* reported for the first time the volatile composition of EB, in 1971 [18], several methodological improvements have been introduced in breath analysis. Therefore, nowadays, EB analysis is no longer restricted to the off-line laboratory approaches, often gas-chromatography (GC) hyphenated methodologies, as GC coupled with mass spectrometry (GC-MS), GC coupled with flame ionization detection (GC-FID), GC coupled with ion mobility spectrometry (GC-IMS), and several real-time EB analysis options are available. Real-time EB analysis includes proton transfer reaction mass spectrometry (PTR) and its variations (PTR-MS and proton transfer reaction-time-of flight-mass spectrometry (PTR-TOF-MS)), IMS and IMS coupled with multi-capillary columns (MCC/IMS), the fast flow and flow-drift tube techniques called selected ion flow tube mass spectrometry (SIFT-MS) (reviewed in [30,73,74]). More recently, a plethora of laser spectroscopy approaches and sensors, commonly known as electronic noses (e-noses) (reviewed in [75,76]), have been developed and applied to EB analysis with promising results (in Table 1 are indicated some examples). These real-time options reduce several unnecessary experimental steps related with sampling, storage and pre-concentration of EB. Moreover, most of them are able to deliver high-sensitivity and high-selectivity in the same range of MS methodologies while operating near real time using potentially inexpensive point of care (POC) devices [75]. One of the most notable advantages of these POC devices is in their utilization in large epidemiological studies. These sensor approaches, however, are confined to the characterisation of a very limited number of target VOCs and their ability to simultaneously identify the broad chemical variation of EB VOCs has to be improved. Alternatively, many e-nose approaches rely on pattern recognition and do qualitative characterisation of different classes of volatiles as compared with the devices that give absolute quantification of volatiles. Either quantitative or qualitative characterisation, the target VOCs have to be identified by comprehensive methodologies, usually involving MS detection. Unfortunately, these comprehensive methodologies are very expensive, requiring highly specialized and skilled operators. The search for a reliable technology for breath analysis is still therefore in progress as there is no single methodology available yet to deliver, for instance, the GC-MS resolution in a real-time approach using an affordable point of care (POC) device.

#### 2.2.1. Off-line Analysis: Gas-Chromatography (GC)

GC was the analytical method chosen for the initial studies in breath analysis [18,77,78,79,80] and even today it is one of the preferred methods when coupled to MS. In fact, almost all of the EB VOCs reported so far have been identified and quantified using MS-based methods, most often GC-MS [75]. GC-MS is possibly the most comprehensive and sensitive approach to characterize EB volatile composition, allowing the selective analysis of one or simultaneous analysis of many compounds that may be in the range from ppb to ppt. The GC-MS involves separation of volatilized samples in chromatographic column based on different parameters, such as polarity of the GC column or the boiling point of the sample components (reviewed in [81]). GC-MS system ionizes the target ions, separate them by mass to-charge (m/z) ratios and uses the resolved fragmentation patterns to quantify the amount of each specific VOC in the sample [81]. There are, however, other detection systems that have been coupled to GC for breath analysis, namely FID and IMS. In the first case, VOCs are burned in the FID, producing ions and electrons that can conduct the electric potential and this information is used for detection and eventually quantification. GC-FID generally exhibits a fairly high sensitivity, large linear response range, and low noise. However, the FID detector is mass sensitive and its response is not altered significantly by changes in mobile-phase flow rate (reviewed in [14]). In turn, in the IMS, ions are separated according to their mobility as they travel through a purified gas, in an electric field at the atmospheric pressure. The IMS detector is also selective, allowing the quantification of VOCs in the EB (reviewed in [14,74]). Regardless of the detection method used in a GC analysis, this approach presents, however, some drawbacks, the most notable being the requirement for sample pre-treatment (sampling and pre-concentration). Therefore, a GC analysis is only suitable for indirect sampling and not for real-time analysis, being a time-consuming process (a typical GC-MS analysis can take up to an hour). Thus, analytes loss and degradation, particularly of those of reactive or thermally labile metabolites, and possible contaminations, are the important concerns inherent to the sample pre-treatment that need to be carefully addressed to improve the quality of the data obtained in a GC analysis [24,33,36,45,75].

**Table 1:** Characterisation of selected exhaled breath (EB) volatile organic compounds (VOCs) reported in the literature.

Target VOCs (Putative Biomarkers) (LODs)	Methodology	Sample (Patients/Controls)	Sensitivity/Specificity (%)	Statistical Approach (*Pre-Processing Method*; Classification Method; Performance Measures)	Reference
**Oncologic Diseases**					
**Lung cancer (LC)**					
1-octene	SPME/GC-MS Chemical nanoarrays	72/10	DFA model: 86.0/96.0 Cross-validation: 86.0/88.0	**LDA**; Wilcoxon/ Kruskal-Wallis ANOVA	[82]
isoprene (81.5 ppb), acetone (458.7 ppb), methanol (118.5 ppb)	PTR-MS/GC-MS	285/472	4 compounds: 52.0/100; 15 (or 21) compounds: 71.0 (80.0)/100	Kruskal-Wallis ANOVA	[30]
isoprene (6041 pM), pentane (647.5 pM), heptane (13.5 pM), octane (61.0 pM), styrene (17.9 pM), among 13 VOCs	SPME/GC-MS	36/50	72.2/93.6	*Kolmogorov-Smirnov*; ANOVA, Games Howell post-hoctest; Kruskal-Wallis ANOVA, Dunn’s Post Hoc test; Student *t-*test; *p*-value; PRISM	[83]
2-butanone (1.78–8.38 nM), 2-hydroxyacetaldehyde (0.13–0.77 nM), 3-hydroxy-2-butanone (0.23–1.13 nM), 4-hydroxyhexenal (0.005–0.05 nM)	FT-ICR-MS	97/88	89.8/81.3	Wilcoxon (Minitab)	[39]
formaldehyde (7 ppb)	PTR-MS	17/170	54.0/99.0	**FQDM**; *p*-values from Wilcoxon; ROC; MATLAB (classify.m)	[84]
pentanal (0.001 nM), hexanal (0.010 nM), octanal (0.009 nM), nonanal (0.028 nM)	OFD-SPME/GC-MS	12/12,12	C5: 75.0/95.5; C6: 8.3/91.7 C8: 58.3/91.7; C9: 33.3/95.8	Kruskal-Wallis ANOVA	[85]
ethane	GC-FID	26/14	-	ANOVA with *Bonferroni’s correction* for multiple comparisons	[86]
isoprene (0.095 nM), acetone (0.985 nM), 2-butanone (0.158 nM), ethanol (5.098 nM), acetaldehyde (1.280 nM), pentanal (0.436 nM), dimethyl sulphide (0.270 nM), pentane (0.431 nM)	SPME/GC-MS	31/31,31	-	**PCA,** Mann-Whitney Rank; Kruskal-Wallis ANOVA; post hoc Student-Newman-Keuls; Dunn’s Method	[87]
hexane, methylpentane, o-toluidine, aniline, alcohols, ketones	e-nose, GC-MS	42/18	Good	**PLS-DA**	[88]
styrene, decane, isoprene, benzene, undecane, 1-hexene, hexanal, propyl benzene, 1,2,4-trimethyl benzene, heptanal, methyl cyclopentane	SPME, virtual SAW gas sensor	20,7/15	Good	***ANN***	[89]
isobutene, methanol, ethanol, acetone, pentane, isoprene, isopropanol , dimethylsulfide, carbon disulphide, benzene, toluene	e-nose, GC-MS	14/45; 14/62	71.4/91.9	**PCA**, **CDA**, **SVM**	[90]
VOCs pattern recognition	colorimetric sensors	49,18,15, 20,20/21	Model validation: 73.3/72.4 21 patients: 100/60.0	**Random forest classifier**	[91]
VOCs pattern recognition	e-nose	10,10/10	-	**PCA**, **LDA**, MVA, CVV, *Savitzky–Golay filtering*	[92]
Set of 42 VOCs	gold nanoparticle sensors, SPME/GC-MS	40/56	-	**PCA**	[93]
VOCs pattern recognition	colorimetric sensors	92/137	High, several groups defined	**Logistic prediction model**	[94]
2-hexanone, 3-heptanone; 2,2,4-Trimethyl-hexane	SPME/GC-MS, sensors	12,4,–1	100/80	**LDA**	[95]
VOCs profile	PTR-MS, SPME-GC-MS	220/441, 65/31	variable	-	[30]
**Mesothelioma**					
VOCs pattern recognition	e-nose	38/42	95/88	**PCA**, **LDA**; *Inbuilt Savitzky–Golay filtering*	[96]
cyclopentane (0.40 ng/L), cyclohexane (4.67 ng/L)	TD-GC-MS	13 + 13/13	92.3/82.7	**PCA, DFA and CP-ANN**; ANOVA	[97]
VOCs pattern recognition	e-nose	13,13/13	92.3/82.7	**PCA**, **DA**; MVA	[98]
**Breast Cancer (BC)**					
nonane; 5-methyl-tridecane; 3-methyl-undecane; 6-methyl-pentadecane; 2-methyl-propane; 3-methyl-nonadecane; 4-methyl-dodecane; 2-methyl-octane	TD-GC-MS	51/102	94.1/73.8	positive predictive value and negative predictive value	[99]
undecane, dodecane, tridecane, tetradecane, pentadecane, d-limonene	TD-GC-MS	54/204	78.5/88.3	ROC, MCCV, MVA algorithm employing WDA	[100]
3,3-Dimethyl-pentane, 5-(2-Methylpropyl)-nonane, 2,3,4-Trimethyl-decane, 2-Amino-5-isopropyl-8-methyl-1-azulenecarbonitrile, 1-Iodo-nonane	GC-MS	22/22	-	**PCA** and **cluster analysis**	[101]
hexanal (3.75 ppbV), heptanal (3.22 ppbV), octanal (3.39 ppbV), nonanal (2.49 ppbV)	GC-MS	22,17/24	72.7/91.7	**Fisher DA; leave-one-out (LOO) DA**; Kruskal-Wallis ANOVA, ROC, AUC	[102]
VOCs profile (**A**: BC on biopsy/normal screening mammograms (scr mam), **B**: normal/abnormal scr mam, **C**: BC/no BC on biopsy)	POC device (TD-GC-SAW)	37 + 35/172	**A** (81.8/70), **B** (86.5, 66.7), **C** (75.8, 74.0)	C-statistic ([AUC] of [ROC]), MCCV, MVA algorithm cross validated with a LOO method	[103]
VOCs pattern recognition	e-nose	16,13/7	94/80	**PCA**, **SVM**, **cross validation**	[104]
**Colorectal cancer (CRC)**					
decanal; 1,3-dimethylbenzene; 1,2-pentadiene Cyclohexane; Methyl cyclohexane; 4-methyloctane	GC-MS	37/41	86/83	**PNN** validated by the **LOO method**	[27]
10 discriminant VOCs	SPME/GC-MS	20/20	-	**PCA**, **PLS-DA**	[45]
4 discriminant VOCs	GC-MS	26/22	-	**PCA** and **cluster analysis**	[101]
**Gastric cancer**					
6 discriminant VOCs	sensors, GC-MS	37,32, –61	89/90	**LDA**; Wilcoxon/Kruskal-Wallis ANOVA	[105]
**Head-and-neck cancer**					
8 discriminant VOCs	e-nose, GC–MS	22,25/40	100/100	**PCA** with ANOVA and Student	[106]
VOCs pattern recognition	e-nose	36/23	90/80	Logistic regression, ROC	[107]
**Liver cancer**					
2,3-dihydro-benzofuran, methane-sulfonyl chloride; acetic acid; ethanol	sensor, GC-MS		95.8/100	**LDA**; Shapiro-Wilk, Wilcoxon/Kruskal-Wallis ANOVA	[108]
hexanal; 1-octen-3-ol; octane	SPME/GC-MS	18/19	100/100	RSD; χ^2^	[109]
3-Hydroxy-2-butanone, styrene, and decane (set A: HCC patients/normal controls; B: cross-validation)	GC-MS	30/27 + 36	A: 86.7/91.7 B. 83.3/91.7	ROC and DA using the defined markers	[110]
**Pulmonary Diseases**					
**Airways inflammation**					
VOCs pattern recognition	e-nose	110/108	72.2/75.1	**k-NN voting rule to classify features extracted by PCA**	[111]
**Asthma**					
Several discriminant VOCs, including acetone and many alkanes	e-nose, GC-MS	20/20	-	**PCA**; **cross-validation** value, **LDA** on principal component reduction, M-distance	[112]
decane; dodecane; tetradecane; 2-methyl-1,3-butadiene; 2,2-dimethylhexane; 2,4-dimethyloctane, 2,3,6-trimethyldecane	GC-MS	35/15	-	**PLS-DA**; Single factor ANOVA	[113]
nonane; 2,2,4,6,6-pentamethylheptane; decane; 3,6-dimethyldecane; dodecane; tetradecane	GC-MS	32/27	96/95	**PLS-DA, Monte Carlo cross-validation (MCCV) statistics**	[114]
Several discriminant VOCs	GC-MS	63/57	89/95	**Stepwise DA; 20-fold CVV DA**	[115]
VOCs pattern recognition	e-nose/GC-MS	27/24	High, several groups defined	**PCA**, *ANN*	[116]
17 discriminant VOCs	GC-TOF-MS	252	high	**Random Forests (RF)** and **dissimilarity PLS-DA**	[117]
**Acute Respiratory Distress Syndrome (ARDS)**					
octane, acetaldehyde and 3-methylheptane	GC-MS	23/53	90	Kruskal-Wallis ANOVA (continuous variables), χ^2^ (categorical variables)	[118]
acetone, isoprene, n-Pentane	GC-FID/GC-MS	19/18	-	Mann-Whitney U-Wilcoxon rank sum test (unpaired samples), Wilcoxon matched-pairs signed-ranks test (paired samples)	[119]
**Pulmonary embolism**					
VOCs pattern recognition	e-nose	40/20	85/65	**LDA**, **PCA**, ROC	[120]
**Pulmonary Tuberculosis**					
6 discriminant VOCs	GC/MS	42/59	95.7/78.9	*Fuzzy logic*, *Pattern recognition analysis*; **PLS, HCA, PCA, k-NN**; PC regression, ROC, SIMCA	[121]
VOCs pattern recognition	POC device (TD-GC-SAW)	130/121	71.2/72	**MCCV**, **multivariate predictive algorithm**; ROC	[122]
Alkanes and derivatives, cyclohexane and benzene derivatives	GC/MS	226	variable	**MCCV**	[123]
**Chronic Obstructive Pulmonary Disease (COPD)/Emphysema**					
Ethane (No steroid treatment—2.77 ± 0.25 ppb; Steroid-treated—0.48 ± 0.05 ppb)	GC-FID	22/14	-	*p-value*; ANOVA—two-way variance analysis	[124]
MDA (57.2 nM), hexanal (63.5 nM) heptanal (26.6 nM)	LC-MS/MS	20/12,20	-	*p-value*; Wilcoxon, Bland-Altman	[125]
Mass-spectra	PTR-MS	-	43/161	bootstrapped stepwise forward logistic regression	[126]
VOCs pattern recognition	eNose	33/10	100/100	**LDA**; Wilcoxon, k-fold cross-validation	[127]
VOCs profile	MCC/IMS	High, variable with statist. used	30 + 54/35	*decision tree, naive Bayes, linear support vector machine (SVM), ANN, RF and radial SVM*	[128]
**Cystic Fibrosis (CF)**					
pentane (0.36 ppb), dimethyl sulphide (3.9 ppb)	GC-MS	20/20	-	Wilcoxon; linear regression	[129]
carbonyl sulphide (110 ± 60 pptv), dimethyl sulphide (4.780 ± 1.350 pptv), carbon disulphide (26 ± 38 pptv)	GC-MS	20/23	-	Student; F-score method; Pearson; Fisher’s z-score	[130]
ethane (no steroid treatment—1.99 ± 0.20 ppb; steroid treatment—0.67 ± 0.11 ppb)	GC-FID	23/14	-	ANOVA with *Bonferroni’s correction*	[131]
**Other Diseases**					
**Cardiovascular Diseases (CVDs)**					
**Acute decompensated heart failure (ADHF)**					
acetone (256–1974 ppb), pentane (20–74 ppb)	SIFT-MS	25/16	-	MVA	[28]
acetone (3.7 ppb)	GC-MS	59,30/20	83/100	Kruskal-Wallis ANOVA	[132]
**Cholesterol**					
Isoprene	GC/MS, SIFT-MS		-	-	[29]
**Atherosclerosis**					
trimethyl amine	GC, SIFT-MS		-	-	[29]
**Carbohydrate malabsorption/maldigestion**					
Ethanethiol, dimethylsulfide	PTR-MS		-	-	[133]
**Liver dysfunctions**					
**Liver Cirrhosis**					
2-butanone (3.2 ± 0.5 ppbv), methanol (528 ± 218 ppbv), heptadienol (2.5 ± 1.4 ppbv), monoterpenes (6.7 ± 5 ppbv)	PTR-TOF-MS	12/14	83/86	*p-value*; **DA**; Wilcoxon, Pearson	[5]
**Non-Alcoholic Fatty Liver Disease (NAFLD)**					
acetone (71.7 ppb), isoprene (14.7 ppb), trimethylamine (5 ppb), acetaldehyde (35.1 ppb), pentane (13.3 ppb)	SIFT-MS	37/23	-	-	[134]
**Alcoholic hepatitis (AH)**					
2-propanol, acetaldehyde, acetone, ethanol, pentane, trimethylamine	SIFT-MS	40,40/43	90/80	*p-value*; Kruskal-Wallis ANOVA, Pearson χ^2^, Spearman correlation	[135]
**Propionic acidaemia**					
3-heptanone	PTR-MS and GC-MS	-	-	-	[133]
**Diabetes mellitus**					
acetone	SPME/GC-MS, SIFT-MS, laser spectroscopy	-	-	-	[29]
acetone	e-nose, SIFT-MS	8	-	-	[136]
acetone (160–862 ppb)	SIFT-MS	-	97.9/100	*p-value*; **Non-parametric tests**	[137]
acetone; isopropanol; toluene; m-xylene; 2,3,4-trimethylhexane; 2,6,8-trimethyldecane; tridecane and undecane	SPME/GC-MS	48/39	-	**PCA**, **OPLS-DA**; MVA, Wilcoxon	[138]
VOCs pattern recognition	e-nose	117/108	87.7/86.9	**k-NN voting rule to classify features extracted by PCA**	[111]
**Chronic renal failure**					
NO (39 ppb)	Ozone chemioluminescence	40/28	-	*p-value*; **DA**; χ^2^	[139]
TMA (0.33 ppb)	TD-GC-MS	14/9	-	Wilcoxon	[43]
Uraemia	IMS/GC-MS	28 + 26/28		ANOVA, two-sided two-sample Student’s *t*-tests	[140]
VOCs pattern recognition	e-nose	110/108	86.6/83.5	k-NN voting rule to classify features extracted by PCA	[111]
**Crohn’s disease**					
Set A (healthy controls/CD remission)- 6 discriminatory VOCs; Set B (healthy controls/active CD); set C (active CD/remission)- 10 discriminatory VOCs	GC-TOF-MS	725/110	A and B (96/97); C (81/80)	**RF** to the most discriminatory VOCs for the 3 groups; **PCA** on proximity matrix obtained from the RF model.	[141]
***Helicobacter pylori*** **infection**					
^13^C O_2_/^12^CO_2_	Cavity Ring-Down Spectroscopy (NIR)	-	100/100	-	[142]
**Schizophrenia**					
ethane and pentane	TD-GC-MS	28/15	-	-	[143]

Abbreviations: TD—Thermal desorption, AUC—area under the curves, COPD—Chronic Obstructive Pulmonary Disease, CVV—cross-validation method, DA—discriminant analysis; FQDM—Fisher’s Quadratic Discriminant Method, FT-ICR-MS– Fourier transform-ion cyclotron resonance mass spectrometry, GC/MS—gas chromatography-mass spectrometry, k-NN—k-nearest neighbour, LDA—Linear Discriminant Analysis; LOO—leave-one-out method, MCCV—Monte Carlo cross-validation, MVA—multivariate data analysis; OPLS—orthogonal PLS; PCA—principal component analysis; PLS—partial least squares; PNN—probabilistic neural network; PTR-TOF-MS– proton-transfer reaction time-of-flight mass spectrometry, PTR-MS– proton-transfer reaction mass spectrometry, RF- Random Forests, ROC—Receiver operator characteristic; SIFT-MS—Selected ion flow tube mass spectrometry, SIMCA—soft independent modelling of class analogy, SPME—solid-phase microextraction, VOCs—volatile organic compounds, WDA—weighted digital analysis.

#### 2.2.2. Real-Time Analysis

Online real-time analysis offers several advantages as compared with indirect sampling, notably yield of immediate results and no requirement for collection and storage of samples. This eliminates a major source of experimental errors in EB analysis, particularly those related with the loss of compounds by different mechanisms (for instance, labile compounds that decompose before being analysed or compounds that change rapidly as a function of external influence [24]). Furthermore, when using MS-based methodologies, as PTR-MS, PTR-TOF-MS and SIFT-MS, it is easier to sample both the EB and the environmental air in the collection room, thus filtering from the results those arising out of exogenous contaminants [32,144]. However, direct sampling also presents some disadvantages such as high cost of acquisition and maintenance of the equipment used, particularly PTR-MS and PTR-TOF-MS. On the other hand, as pre-concentration of EB is not possible, certain low abundant VOCs cannot be detected by this approach [32,144].

##### 2.2.2.1. Proton Transfer Reaction Mass Spectrometry (PTR-MS)

PTR-MS use in breath research is relatively recent, but very promising because it can deliver results in a real-time online analysis, with high sensitivities for VOCs detection and quantification (up to the pptv range). This analytical performance compares in terms of sensitivity (but not selectivity) with the one obtained by GC-MS (or is even greater, as shown by Bajtarevic *et al.* [30]). PTR-MS uses H_3_O^+^ ions for proton-transfer reactions with many common VOCs, while having little to no reaction with the highly abundant atmospheric gases (N_2_,CO_2_ and H_2_O) that compose more than 99% of EB (reviewed in [14,73,133]). There are, however, a few disadvantages to point to PTR-MS, namely the number of compounds that can be simultaneously analysed and the limit of detection. According to Herbig *et al.* [144], in an online breath analysis, a minimum of 3 Hz of sampling frequency is necessary in order to resolve individual breath phases. Thus, this trade-off between the number of measured *m/z*, and the signal-to-noise ratio (S/N), limits the number of compounds that can be simultaneously monitored and the respective limit of detection (LOD) that can be obtained. On the other hand, as the PTR-MS detection relies on the atomic mass of compounds and the resolution of quadrupole MS instruments is limited, it is impossible to identify compounds with the same molecular weight [144,145]. To overcome this sensitivity disadvantage, a TOF-MS was associated to the PTR, *viz.* the PTR-TOF-MS. In this technique, similarly to GC-TOF-MS, the ions are accelerated to a regular energy by an electric field. Then the ions travel a defined distance without acceleration. The m/z will determine the time of flight of the compound. Thus, the mass spectrum can be obtained by measuring a single shot (a fraction of a second) or adding more shots (although this last option increases the S/N (reviewed in [144]). This methodological improvement allows a three order of magnitude increment in PTR-TOF-MS and consequently it is now possible to separate distinct chemical compounds with the same molecular weight using this real-time approach ([146] reviewed in [144]). Nevertheless, as already mentioned, since pre-concentration is not possible, very low abundant VOCs can hardly be detected using this approach. Moreover, it is a far more expensive technique than GC-MS [32,144].

##### 2.2.2.2. Selected Ion Flow Tube Mass Spectrometry (SIFT-MS)

SIFT-MS is a technique that combines the fast flow tube technique with MS, allowing a real-time measurement of trace concentrations of VOCs in humid air, including EB. In simple terms, EB VOCs are collected into the flow tube and ionized with precursor ions (usually H_3_O^+^, NO^+^, or O_2_^+^), thereby forming the product ions, which are then quantified by MS. A detailed review and comparison of the methodology with PTR-MS can be found elsewhere [3,14,73].

##### 2.2.2.3. Ion Mobility Spectrometry (IMS)

IMS was initially developed for the high sensitive detection of chemical warfare agents, illegal drugs and explosives, and adapted to industrial and environmental applications, particularly for process control in food quality analysis and air quality control. In simple terms IMS uses an external electric field at ambient pressure to separate different ions formed from the target analytes. This is achieved in different ways, using differential mobility spectrometers (DMS), high-field asymmetric waveform ion mobility spectrometers (FAIMS), homemade IMS or commercially available IMS, such as without and with different gas chromatographic columns, e.g., MCC (multi-capillary column)/IMS. Overall, the sensitivities in the ppbv- to pptv-range that can be achieved with IMS have made it suitable for breath analysis. In fact, different IMS strategies have been successfully used in the medical field, particularly in the diagnosis of several pulmonary diseases, such as lung cancer, COPD, lung infections and asthma, as well as other bacterial infections (reviewed in [74]).

#### 2.2.3. Targeted Breath Analysis

Regardless of its utilization off-line and online, MS-based approaches for EB research enable a comprehensive analysis that is mandatory for the characterisation of new putative biomarkers. However, the requirement of expensive equipment and high levels of expertise to operate them constitute two important barriers to their use as POC devices. Therefore, a number of other approaches based on laser-absorption spectroscopy and chemical sensing are being developed with promising results (reviewed in [14,75,76,81,147]) and used in different POC devices generically known as electronic noses or “e-noses”.

##### Electronic Noses (e-noses)

In a very recent report, Bushdid *et al.* demonstrated that the human olfactory system can discriminate at least 1 trillion olfactory stimuli [12]. This reveals an extraordinary in-built metabolomics in mammalians that are able to discriminate a broad range of odours by VOCs patterns. A similar approach is used in many e-nose devices. In this case, the objective is to mimic the human olfactory system in the recognition of odours as “smellprints” or VOCs patterns of different disease conditions. This is quite different of the identification and quantification of individual VOCs present in a mixture, as performed by the approaches described in the previous sections. It should be highlighted, however, that most of the first e-noses to be developed rely only in the identification of a specific compound or a class of compounds, particularly key VOCs in EB as isoprene, CO, NO, alkanes, *etc.* (see Table 1). Their main advantage is being POC devices suitable for an eventual utilization as medical device. Overall, e-noses use GC, IMS and MS, optical sensing or infrared spectroscopy (usually near infrared spectroscopy, NIR) and a generic architecture comprehending a multiple sensor array, the data acquisition system, and a pattern recognition algorithm (reviewed in [76,148]). The GC, IMS and MS approaches are based on the same principles as the corresponding bench-top technologies, but using alternative building materials and operating modes ([149,150,151]). The optical sensors use diverse light sources for measuring changes in a given light property when it crosses the gas mixture (reviewed in [76]). Among the optical sensors, the laser spectroscopy approach is very popular. In this case, a laser beam is used against a gas mixture, as EB samples. As many target analytes absorb in a highly specific wavelength, often referred as spectral fingerprint, the amount absorbed can be easily measured in a detector and correlated to the target analytes concentration [75]. As already referred, laser spectroscopic detection techniques are able to deliver MS-equivalent high-sensitivities and selectivities, in an online and real-time fashion response and using simple and relatively inexpensive POC devices. In fact, 14 out of 35 putative breath biomarkers (acetone, ammonia, CO_2_, ethane, methane, and NO, among others) have been already analysed by laser spectroscopic techniques (reviewed in [75]). The application of these e-nose solutions to large populations is much easier than a comprehensive study using MS detection and certainly the data obtained would be very relevant. Nevertheless, there are a number of issues that have to be solved before e-noses can reach a level to be used in medical diagnosis (reviewed in [7,152,153]).

## 3. The Metabolics of EB Volatiles

Every disease has associated alterations in the normal physiology and metabolism and are characterised by some changes at the level of gene regulation, proteins expression and metabolites production. Some of these changes can be disease specific and hence could be used as “biosignatures” for those diseases. In this sense, the occurrence of certain VOCs in EB as a result of alterations in various metabolic pathways can distinguish a disease state from a healthy state. These volatile metabolites are majorly consisting of inorganic gases, like nitric oxide (NO) and carbon monoxide (CO), and VOCs as hydrocarbons (pentane, ethane and isoprene), oxygen-containing compounds (acetone, acetaldehyde, methanol, ethanol, and 2-propanol), sulphur-containing compounds (dimethylsulfide, methyl, and ethyl mercaptanes), and carbon disulfide and nitrogen containing substances like ammonia and dimethyl/trimethylamine (reviewed in [154]). Overall, these and other VOCs allow insights into different biochemical pathways involved in various diseases and sometimes are even considered as putative biomarkers of those diseases. In this section we will address the possible biochemical metabolic mechanisms of most frequent VOCs reported in EB. In the end, we will explore the potentialities of breath analysis in the diagnosis of different diseases (Table 1).

### 3.1. Hydrocarbons

#### 3.1.1. Saturated Hydrocarbons

Lipid peroxidation is a hallmark of several diseases and clinical conditions, such as cancer, inflammatory diseases, atherosclerosis, and aging [31] and hydrocarbons, such as ethane and pentane, are the end products of this highly deleterious reaction. Lipid peroxidation is a chain reaction triggered by removal of allylic hydrogen atoms upon reactive oxygen species (ROS) attack. This results in the formation of very reactive peroxides that participate in further oxidative reactions, particularly against lipids of the cell membrane, viz., ω3 and ω6 fatty acids. As a result, saturated hydrocarbons, such as ethane and pentane, are generated from ω3 and ω6 fatty acids, respectively [154]. Malondialdehyde (MDA), another aldehyde, is also formed in the same pathway. Both *in vitro* and *in vivo* studies have shown a very similar pattern in diseases involving high peroxide activity and release of ethane and pentane [155]. Therefore, these aliphatic hydrocarbons are considered as biomarkers for both *in vitro* as well as *in vivo* lipid peroxidation processes [155]. And although these VOCs can be generated by some colonic bacteria via metabolism and protein oxidation, their contribution does not correlate with the EB assessment for ethane and pentane under situations in which high oxidative conditions are present [156]. Nevertheless, there are some additional metabolic considerations for ethane and pentane. The physiological ratio of ω3 and ω6 fatty acids is in such a way that the amount of pentane that results from lipid peroxidation is four times higher than ethane. Pentane, however, is easily metabolized by cytochrome P450 enzymes present in hepatocytes [157]. Therefore, in the interpretation of EB pentane concentration, a situation of liver function variability has to be carefully taken into consideration during the sampling period and between the patients. In a study by Dryahina *et al.* on inflammatory bowel disease, pentane was found to be significantly elevated in the breath of both the Crohn’s disease and ulcerative colitis patients as compared to the healthy individuals [158]. Overall, pentane and ethane abundances are increased in inflammatory diseases [154] as well as in lung cancer [75]. Hydrocarbons are stable end products of the process of lipid peroxidation and have low blood solubility as a result of which they are diffused into the breath within a few minutes of their generation inside the tissues. Therefore, the concentration of exhaled ethane and pentane can become a useful monitoring approach for the extent of oxidative damage in the body [159,160]. Regarding this, Ross *et al.* used breath ethane and pentane to show that unmedicated patients with schizophrenia already exhibit elevated levels of oxidative stress and this is not a consequence of the medication these patients usually take [143]. Data about other saturated hydrocarbons is scarcer. Propane and butane, for instance, seem to be exclusively formed by protein oxidation process and gut flora and their importance as markers of lipid peroxidation is unclear [154]. In turn, Bos *et al.* reported an increased concentration of octane (together with acetaldehyde and 3-methylheptane) in Acute Respiratory Distress Syndrome (ARDS) [118].

#### 3.1.2. Unsaturated Hydrocarbons

##### Isoprene

2-methyl-1,3-butadiene commonly known as isoprene (unsaturated hydrocarbon) is a major constituent of EB [161], being easily detectable at ppb range [162,163,164]. There is little information available about the biochemical source of isoprene, although it is known that the compound has an endogenous origin [77,161,165,166,167,168,169,170], and it is not a product of the airways [171]. A growing number of evidences pointed out isoprene as a by-product of cholesterol biosynthesis along the mevalonic acid pathway [166,167,168,172,173,174,175,176,177]. One of the key steps for the cholesterol biosynthesis is the synthesis of mevalonic acid from acetic acid [174] which is a rate limiting step for the synthesis of sterol and is catalysed by hydroxymethylglutaryl coenzyme-A (HMG)-CoA. Mevalonic acid is further transformed into isopentenyl pyrophosphate inside the cytosol, which further goes through isomerization to form dimethylallyl pyrophosphate (DMPP) [178]. With the help of a carbonium ion intermediate generated in the previous steps, DMPP is quickly converted to isoprene by an acid-catalysed elimination reaction in liver cytosol [167]. It is still unknown whether this particular non-enzymatic reaction actually results in the formation of isoprene under the physiological conditions. In some plants, this reaction (the production of isoprene from DMPP) is catalysed by a Mg^2+^ containing enzyme [176]. Therefore it is possible that a similar enzyme may be responsible for catalysing the isoprene conversion from DMPP in the mammalian tissue. The candidate is the Mg^2+^-dependent isopentenyl pyrophosphate isomerase. The involvement of isoprene cholesterol metabolism is further supported by the observation that there is a simultaneous decrease in isoprene secretion and sterol synthesis upon the administration of lovastatin, a lipid-lowering drug. This strongly suggests that isoprene found in breath is derived from the cholesterol synthesis pathway in humans. Moreover, the administration of Lovastatin to healthy individuals and ICU (intensive care unit) patients showed a proportional decrease in isoprene as well as plasma total cholesterol concentrations [174]. Additionally, a cholesterol-rich diet is also responsible for decreased levels of isoprene in exhaled breath [174] because of the feedback inhibition of HMG-CoA reductase (3-hydroxy-3-methyl-glutaryl-CoA reductase) [179,180]. Isoprene concentrations correlated with cholesterol biosynthesis [75,154] and hence the monitoring of breath isoprene may be helpful as a marker for the lipid and cholesterol status monitoring and even in analysing the efficiency of lipid-lowering therapy. There is, however a strong positive correlation between muscle activity and isoprene [181,182] and this constitutes a major confounder for any correlation with cholesterol metabolism. Nevertheless, Isoprene has also been studied in other diseases. In lung cancer, isoprene shows a decreased concentration [30]. In another study on lung cancer patients, breath isoprene levels correlated quite significantly with increased total cholesterol and LDL, although no significant relationship has been observed between isoprene and HDL, triglycerides and C-reactive protein [183]. Breath isoprene is both age dependent, with males typically presenting higher levels than females, and affected by a circadian rhythm with levels peaking between 2 AM. and 6 AM (reviewed in [29]). Overall, it is widely accepted that breath isoprene reflects the rate of cholesterol production, although there are not enough studies to verify this correlation with the degree of cholesterolaemia and evaluate the interference of the contribution of muscle activity for isoprene levels.

### 3.2. Ketones

#### 3.2.1. Acetone

**Figure 3:**
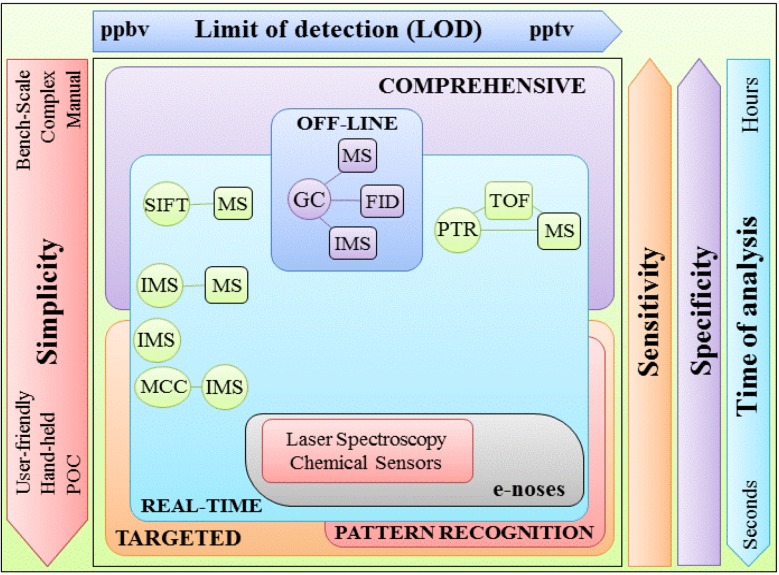
Overview of the methodologies most used in exhaling breath (EB) analysis and comparison of some analytical features, as limits of detection, sensitivity and specificity. Additional inputs as time of analysis and simplicity are also included. Abbreviations used: FID—flame ionization detection; GC—gas chromatography; MCC-IMS—multi capillary column ion mobility spectrometry; POC—point of care; PTR-TOF-MS-proton transfer reaction with time-of-flight mass spectrometry; SIFT-MS—Selected ion flow tube mass spectrometry.

Acetone is one of the most notable ketone produced by the human metabolism and also a major constituent of human breath. Different mechanisms can lead to its formation. It is absorbed in the body from exogenous sources of contamination, such as food, effluents from chemical industries. Endogenously, acetone can be produced in the hepatocytes through the decarboxylation of excess Acetyl-CoA (Figure 3), but any metabolic change involving an increase in fatty acid oxidation (exercising, fasting, food consumption, weight loss, cancer, and also protein metabolism) results in the formation of ketone bodies, like acetoacetate, β-hydroxybutyrate and acetone. In turn, the spontaneous decarboxylation of acetoacetate can also yield more acetone. Remarkably, acetone is the ketone body produced in smaller quantities. Its higher volatility, however, make it easily detectable from breath, urine and skin. In the uncontrolled diabetes mellitus patient’s breath, for instance, acetone concentrations are particularly elevated, constituting an easy way to assess the treatment success and a supporting disease monitoring approach, when combined with the regular glucose level checking [184,185,186]. In this context, Wang *et al.* also reported that patients with type 1 diabetes have excess acetone in their breath [75]. In turn, Storer *et al.*, studied type 2 diabetic patients undergoing a long term dietary modification programme and observed that breath acetone concentrations vary between 160 and 862 ppb [137]. Nevertheless, acetone can not be considered as a marker of diabetes mellitus, but instead of ketone bodies formation. In turn, ketone bodies are a measure of blood glucose levels. Acetone variations have been described in many other diseases and clinical conditions. Alkhouri *et al.*, for instance, studied overweight and obese children with Non-Alcoholic Fatty Liver Disease (NAFLD) and found many VOCs, including breath acetone, in significantly higher concentrations [134]. In another study, on liver disease, Hanouneh *et al.* were able to distinguish between patients with Alcoholic Hepatitis (AH), acute decompensation and individuals without liver disease using several breath metabolite levels, mainly breath acetone [135]. Marcondes-Braga *et al.* found higher EB acetone concentrations in Heart Failure (HF) patients as compared to healthy control volunteers. In this study, they exhaustively monitored the condition of HF in a large sample size and reported breath acetone as a new biomarker of HF severity [132]. Overall, given the several sources and situations that can lead to an increase in the concentration of EB acetone, this ketone can hardly be considered as a biomarker of a particular disease or clinical condition. Nevertheless, taking together with additional parameters, it provides very relevant information about the human metabolism.

**Figure 4:**
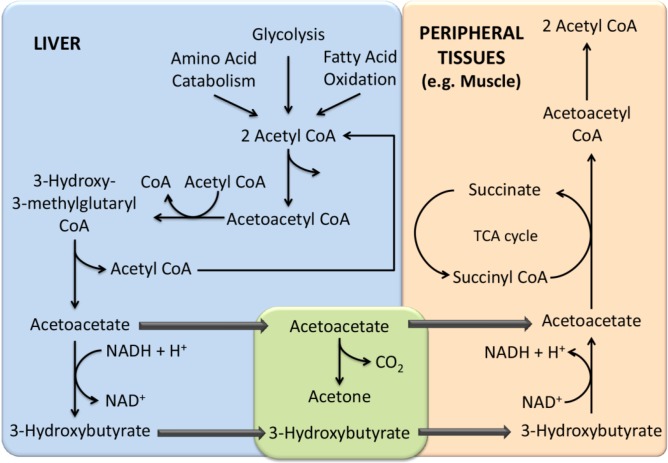
Metabolism of acetone and its precursors in liver and muscle.

#### 3.2.2. 2-Butanone

2-Butanone is an important ketone, being widely used as a raw material in different industries, such as the paints, coatings, resins, and printing industries. For a long time it has been associated with some toxicity, but since 2005, it has been reclassified as a non-hazard air pollutant [187]. 2-Butanone occurs naturally in the environment, although in negligible levels when compared with its industrial demands, being secreted from many different species of bacteria, fungi, and plants ([188,189,190,191,192,193,194]). Remarkably, it was recently shown to elicit induced acquired resistance in the cucumber against two natural predators [195]. 2-Butanone seems to be ubiquitously present and has been detected in several human fluids of apparently healthy volunteers, including skin emanations, saliva, urine, faeces, blood and exhaled breath ([196,197,198,199]). In a recent review about lung cancer VOCs and their possible biochemical pathways, Hakim *et al.* classified 2-Butanone as dietary and environmental contaminant [16]. However, several other studies reported the augmented presence of 2-Butanone in the breath of patients with different diseases conditions, including liver cirrhosis [5], lung cancer [87,200,201], ovarian cancer [202], *H. pylori* infection [203] and sepsis [204]. Moreover, Fu *et al.*, were able to discriminate lung cancer patients in different stages through 2-butanone, showing that its concentrations were significantly higher in the exhaled breath of non-small cell lung cancer (NSCLC) patients in stages II though IV when compared to with stage I [39].

### 3.3. Nitrogen Containing Compounds

#### 3.3.1. Nitric Oxide (NO)

NO is the most important nitrogen compound in terms of volatile EB biomarkers, being produced by different types of cells in the respiratory tract, such as inflammatory, epithelial, and vascular endothelial cells and airway nerves [205]. NO has one unpaired electron, which makes it highly reactive towards other molecules. It has a half-life of around 1–5 seconds and diffuses freely across membranes. These properties make NO an important transient signalling molecule working both within a single cell (autocrine) and between adjacent cells (paracrine) [206]. In fact, NO modulates the endothelium vasodilatation activity and for this reason is also termed as the endothelium-derived relaxing factor, EDRF. It is used by the cells to send signals to the surrounding smooth muscle to relax, thereby, causing vasodilation and blood flow increase. This physiological activity is influenced by several factors, as the levels and activity of the enzymes involved in NO production, its uptake rate by antioxidant molecules (glutathione and haemoglobin, for instance), and the levels of oxidative stress [207]. Additionally, NO can be generated in the phagocytes (macrophages, neutrophils, and monocytes) that use it against pathogens (human immune response). Phagocytes express an inducible NO synthase (iNOS) that is regulated by interferon-gamma (IFN-γ) and tumour necrosis factor (TNF) (activators) [208,209,210], transforming growth factor-beta (TGF-β) (strong inhibitors) and the interleukin-4 (IL-4) and IL-10 (weak inhibition). This tight regulation of iNOS reflects the importance of NO in inflammation and immune responses [211]. In fact, NO is distinctively observed in the bronchial system and is reported to be a marker for airway inflammation [154]. This assumption results from extensive studies in different lung diseases, particularly those in which NO metabolism is altered. Regarding this, Ghosh *et al.*, for instance, suggested that high NO levels are present in the asthmatic airway environment, which is oxidative in nature. This further leads to an increase in the generation of reactive nitrogen species (RNS) and consequent protein nitration and oxidation. Ultimately, these modifications adversely affected protein functions, contributing to chronic inflammation [212]. Therefore, NO, through the measurement of its fractional concentration in exhaled breath (FENO), has become an important example of an exhaled biomarker that has reached clinical practice, particularly in the management of asthma [213,214]. This pulmonary complication is characterised by recurrent and reversible airflow obstruction with different degrees of severity [215] and FENO helps not only in its diagnosis, as well as in the discrimination of its phenotypes and treatment monitoring, [216,217], being a surrogate marker of eosinophilic airway inflammation and good predictor of corticosteroid response [218]. Overall, FENO constitutes a valuable diagnostic tool for a reliable clinical guidance in asthma treatment, particularly when its values are low [219]. Consequently, several devices using chemiluminescent and electrochemical sensors able to accurately measure FENO are already present in the clinical environment and other approaches, using quantum cascade lasers, for instance, are being developed [220,221]. Chronic obstructive pulmonary disease (COPD), is characterised by a generalized inflammation that causes airways obstruction and, consequently, breath shortness [222]. In this case, NO has been correlated to the severity of COPD and higher levels are found in unstable COPD patients [223]. Similarly, promising FENO discriminative variations between patients and controls are observed in other complications besides COPD and asthma, including cystic fibrosis (CF), systemic sclerosis, hepatopulmonary syndrome and primary ciliary dyskinesia [224,225]. Additionally, Fisher *et al.* also reported increased exhaled NO levels in lung-transplant recipients with lymphocytic bronchiolitis and early obliterative bronchiolitis [226]. These findings suggested a possible role for exhaled NO also as a marker of pulmonary allograft dysfunction (reviewed in [14]). FENO can become also a useful diagnostic tool in several non-pulmonary diseases, namely CVDs. In this context, Baylis reported that the overall NO production is reduced in chronic kidney disease (CKD), promoting cardiovascular deleterious modifications and further kidney damages [227]. Using an ozone chemiluminescence technique, Matsumoto *et al.* also found higher exhaled NO concentrations in chronic renal failure patients [139]. In turn, Huang *et al.* reported for the first time the presence of exhaled NO along with other metabolites in the exhaled breath condensate (EBC) of Paediatric Inflammatory Bowel Disease (IBD) patients [228].

#### 3.3.2. Dimethylamine (DMA) and Trimethylamine (TMA)

The higher levels of nitrogen containing compounds, such as DMA and TMA, are the cause of the distinctive odour of the breath of uremic patients. The identification and quantification of these amines was carried out way back in early twentieth century, when a study with chronic kidney disease patients showed that the occurrence of TMA in their breath could be considered as a potential marker of renal disorder, previously detected in plasma [43]. In uremic breath patients, ammonia also has a distinctive presence. In normal conditions, ammonia is eliminated via conversion to urea, but in the presence of liver function abnormalities, this elimination is not totally fulfilled and higher levels of ammonia remain in the blood. As ammonia is highly volatile, it can be then easily detected in these uremic patients breath [229].

#### 3.3.3. Acetonitrile (ACN)

One of the most important nitrile compounds in human breath is ACN, which is a key component of tobacco smoke. This has been shown in several studies reporting the higher breath ACN concentrations in smokers as compared with non-smokers [84,87]. A similar observation was also reported in lung cancer patients who were ex-smokers when compared with lung cancer smokers [30] and healthy smokers [87]. The pathway behind ACN metabolism is its biotransformation by cytochrome p450 monooxygenase to cyanohydrine, and its further spontaneous decomposition into hydrogen cyanide and formaldehyde. ACN metabolism, as reported by Li *et al.* [230] is quite slow, hence a significant part is excreted as ACN in the EB and urine [231]. These authors used Extractive ElectroSpray Ionization Mass Spectrometry (EESI-MS) to follow EB ACN concentration in active and passive smokers, and non-smokers. They concluded that the concentrations of EB ACN increases continuously for 1–4 h after the smoker finished smoking and then slowly decreases to the background level in 7 days [230].

### 3.4. Aldehydes

Aldehydes participate in the human metabolism through different forms, being involved in signal transduction, gene regulation, and cellular proliferation, although some of them are cytotoxic intermediates [232,233]. These compounds can be generated in the body through various mechanisms, though metabolic conversion of alcohols being their major source (reviewed in [234]). Another mechanism for aldehydes generation is the reduction of hydroperoxide by cytochrome p450 as a secondary product of lipid peroxidation. The hydroperoxy bond undergoes a stepwise one-electron reduction, in which the first reductive step yields an alkoxy radical. This radical undergoes the well-known β-scission reaction to yield a ketone or an aldehyde and a derived radical, R′^•^ [235]. The endogenous production of aldehydes can be also caused by the smoking habit, that generates both saturated compounds, such as formaldehyde, acetaldehyde, propionaldehyde, butyraldehyde, and unsaturated compounds, such as acrolein and crotonaldehyde [236]. Additionally, smoking can generate aldehydes inside the body as by-products of tobacco metabolism mediated by cytochrome P-450 during the detoxification process [237]. Regarding this, 4-(methylnitrosamino)-1-(3-pyridyl)-1-butanone (NNK) is a major constituent of tobacco smoke and a known carcinogenic agent (a detailed mechanism of NKK carcinogenic effects can be found elsewhere [238,239,240]). Overall, regardless of the specific mechanism involved in their formation, many aldehydes have been described as promising biomarkers in different diseases, as reported, for instance, by Fuchs *et al.* studying lung cancer patients [85].

#### 3.4.1. Formaldehyde

Formaldehyde is metabolized from methanol by the enzyme alcohol dehydrogenase (ADH), which is also involved in ethanol conversion to acetaldehyde. The same enzyme can then oxidize these endogenous aldehydes, yielding carboxylic acids. Wehinger *et al.* reported higher formaldehyde concentration in the breath of lung cancer patients compared to healthy individuals [84]. The same study supported the finding that formaldehyde is generated by lung cancer and immune cells, while tryptophan degradation is taking place during metabolism [241] resulting in the impairment of the function of immune system during carcinogenesis [242,243]. In another study involving lung cancer patients, healthy smokers and healthy non-smokers, formaldehyde concentration was found elevated in lung cancer patients and healthy non-smoker breath as compared to healthy smoker breath [85]. Finally, formaldehyde has also been reported to be augmented in breast cancer patients [75]. Formaldehyde has a high solubility in water and is almost entirely absorbed in the respiratory system when inhaled from the environment. To cope with its toxicity, the formaldehyde dehydrogenase (FDH, an ALDH of the respiratory tissues), oxidizes formaldehyde after binding to glutathione (GSH), forming the adduct S-hydroxymethylglutathione.

#### 3.4.2. Hexanal and Heptanal

Hexanal and heptanal are another two aldehydes observed in urine, breath, and blood samples of lung cancer patients [85,102,244]. Their levels were also reported to be augmented in patients with stage I Non-Small Cell Lung Carcinoma (NSCLC) [82,85,244]. Further comparison of their breath and blood concentrations, suggested that breath hexanal and heptanal are originated from blood. Therefore, hexanal and heptanal breath screening for lung cancer seems very promising and must be further investigated [244]. Finally, in another study using breast cancer patients, four aldehydes (hexanal, heptanal, octanal, and nonanal) were found to be in significantly higher concentrations when compared to healthy individuals [102].

### 3.5. Potential Use of Breath Analysis in Different Diseases

In this section we briefly discuss current trends in disease diagnosis using EB analysis. Table 1 gives the examples of EB VOCs that form putative biomarkers for different diseases. The suitability of breath analysis for disease diagnose depends firstly on its discriminatory ability to classify patients according to the diseases affecting them. Therefore, the sensitivity and specificity of the methodology has to be robust and there should be very limited number of false negatives and false positives otherwise lives can be in danger due to unnecessary medical treatments and interventions respectively.

#### 3.5.1. Oncologic Diseases

Lung cancer (LC) is the leading cause of cancer mortality, accounting for 28% of the cancer related deaths [30,82,245]. The National Lung Screening Trial (NLST, USA) demonstrated that LC screening by low dose computed tomography (LDCT) scans reduced the lung cancer mortality rate by 20% [39,82]. Currently, CT and bronchoscopy are the principal techniques used for lung cancer detection [39]. The diagnosis of early stage lung cancer is critical for the life expectancy of the patients as its five-year survival is around 58%–73% when lung cancer is diagnosed in stage I, but drops abruptly to only 3.5% for later stages diagnosis. Taking in account that only 15% of lung cancer cases are diagnosed at an early stage, more than half of lung cancer patients die within the first year of upon the diagnosis [245]. The main cause for this is that available diagnostic methods are not sensitive enough and, moreover, they are expensive and invasive. Also for this reason, prescription of these methods for diagnosis often happens during late stages of the disease [30,245]. There are strong evidences suggesting that certain cancers can be detected by molecular analysis of exhaled air [30] and for that reason exhaled VOCs are promising candidates as LC biomarkers. These VOCs are emitted from the membrane of the cancer cells and/or from the surrounding microenvironment to the blood stream [16,82]. The cancer-related changes in the blood chemistry are then reflected in measurable changes to the breath through excretion via the lungs [82]. Previous studies have shown that the breath VOC profile of LC patients differs from healthy subjects. The results of these studies indicated that the breath test might have future potential for the management of patients with pulmonary nodules [82,245]. In fact, monitoring VOCs in the breath may rapidly become an interesting addition or an alternative to conventional medical diagnostics. This innovative approach could transform LC care and management by allowing non-invasive diagnosis, prediction of the metastatic potential of the cancer cells, adapting individual treatment, and monitoring therapeutic success [16,246]. Table 1 gives several examples of EB studies applied to lung cancer using different approaches, including GC [85,86,87,97], PTR-MS [30,84] and several e-nose and sensor devices [82,88,89,90,91,92,93,94,95,96,98]. It is noteworthy to mention here that the colorimetric sensor array developed by Mazzone *et al.* detects volatile, active compounds present in the EB of LC patients [91,94]. Colorectal cancer (CRC) is the second leading cause of cancer-related death in Europe and the third in the USA. The search for new and non-invasive screening system with the potential for high patient compliance and of low cost is still continuing (reviewed in [247]). Two promising studies, however, should be mentioned: Altomare *et al.* compared EB samples from CRC and healthy patients using GC-MS and identified 15 discriminating VOCs [27]. In turn, Peng *et al.* analysed EB samples from patients affected by several forms of cancer using GC-MS and a custom nanosensor array. They were able not only to discriminate between cancer patients and healthy controls (GC-MS), but also between different forms of cancer (CRC, breast, lung and prostate cancers) [101].

Gastric cancer is one of the most common causes of death from cancer worldwide. The lack of defined risk factors and the unspecific clinical symptoms often delay its diagnosis, leading to a poor prognosis and high rates of recurrence. The usual technique for diagnosing gastric cancer is the upper digestive endoscopy combined with biopsy and histopathological evaluation of the biopsy samples. However, this method is invasive, relatively expensive, and requires highly skilled medical staff. Therefore, it is necessary to find a simple and non-invasive alternative to this screening test. A pilot study from Xu *et al.* using a nanomaterial-based breath test identified several VOC differentially present in the EB of gastric cancer patients [105].

Breast cancer (BC) is the most prevalent form of cancer diagnosed in women [248]. Its early diagnosis is very important to reduce the BC mortality rate, but the conventional methods (mainly echography, X-rays and tissue biopsies) are expensive and uncomfortable and invasive for the patient. EB analysis is a promising approach for the early BC diagnosis, but a limited number of studies have been performed to support it [99,100,101,102,103,104].

#### 3.5.2. Pulmonary Diseases

Lung cancer is certainly one of the most important pulmonary diseases to study, but there are several other pulmonary complications that have been assessed using EB analysis. This include, for instance, airways inflammation [111], asthma [112,113,114,115,116,117], acute respiratory distress syndrome (ARDS) [118,119], pulmonary embolism [120], chronic obstructive pulmonary disease (COPD) [124,125,126,127,128], pulmonary tuberculosis [121,122,123] and cystic fibrosis (CF) [129,130,131]. Asthma is an inflammatory disease of the airways and the most prevalent chronic illness in childhood. It’s diagnose is invasive, requiring the collaboration of the patient and therefore its reliability, although high in adults, is very poor in children. Therefore it is very relevant to highlight here the recent work from Smolinska *et al.* that used breath analysis to characterize early predictive signatures of asthma in preschool children (2–6 years) [117]. Their work is particularly interesting because there isn’t available any reliable methodology that can be used on children with confounding symptoms (transient wheezing, cough and difficulties in breathing) and discriminate such children from those who would develop asthma [117]. Another pulmonary complication, pulmonary tuberculosis, is becoming a serious health problem, particularly in the developing countries. The common methods used in the screening of this disease are sputum smears and chest radiographs. These approaches are highly specific for active pulmonary tuberculosis, but their value in primary screening is limited by its low sensitivity and high cost. Breath VOCs might provide new biomarkers for active pulmonary tuberculosis, since, for instance, patients suffer from increased oxidative stress which creates distinct VOCs patterns [121]. Nevertheless, oxidative stress is a hallmark of all inflammatory processes and so additional specific VOCs for tuberculosis diagnose must be found. CF is characterised by a chronic airway inflammation, retention of viscous secretions, bronchiectasis and, often, bacterial infection [129,249]. Most treatment decisions continue to be based on clinical judgement and secondary parameters that derive from pulmonary function testing, chest radiography or blood analysis. Bronchoscopic lavage or biopsy can be applied in the evaluation of inflammatory processes, but risks associated to this invasive medical procedure are often unjustified [129]. Therefore, it is necessary to find biomarkers of inflammation in order to monitor disease progression.

#### 3.5.3. Other Diseases

Several VOCs can be linked directly to cholesterol metabolism, as isoprene. Therefore, its measurement in EB can be used to monitor cholesterol metabolism [29] and eventually cardiovascular diseases (CVDs). Acute decompensated heart failure (ADHF) is the most common cause of hospitalization, especially at the elder age. However, the identification of the individuals with imminent decompensation using the conventional clinical methods is unreliable. Some studies have reported elevated levels of acetone, pentane, and NO in the exhaled breath of heart failure patients suggesting the viability of the exhaled breath analysis in ADHF [28,132]. EB analysis has also been used to assess chronic renal failure [43,111,139,140]. Pagonas and his colleagues, for instance, using IMS followed by GC-MS, identified VOCs that are retained in uraemia and observed significant differences between patients with and without renal failure [140]. This result indicates the possible use of breath analysis as a screening method for renal failure. This is particularly interesting for paediatric care due to its non-invasive nature. Additionally, it may be used for real-time monitoring of haemodialysis efficacy [140]. In liver diseases, breath analysis has been used to study hepatocellular carcinoma [108,109,110], cirrhosis [5], non-alcohol fatty liver disease (NAFLD) [134] and alcoholic hepatitis (AH) [135]. Recently, it was also possible to distinguish the breath of patients with Crohn’s disease and patients with active ulcerative colitis, using GC-TOF-MS [141]. Using SIFT-MS, EB pentane was shown to be a putative biomarker of bowel disease [158]. In this case, however, the discriminative ability of pentane as a biomarker of bowel disease should be very limited as alkanes, namely pentane and ethane, are two very promising biomarkers of oxidative stress (reviewed in [182]), which in turn is a hallmark in several diseases, including cardiovascular, oncologic and neurodegenerative diseases. Another very relevant work reports an e-nose composed by an array of sensors for different EB compounds that was able to discriminate patients with diabetes, airways inflammation and chronic renal disease with promising results [111].

## 4. Data Analysis and Discriminatory Models Used in Breath Biomarker Research

The ultimate goal of breath analysis utilization in disease diagnosis, is to identify a set of VOCs that show statistically significant variation between patient and control samples. However, discovery of such a set of discriminatory VOCs suffers from some practical challenges and bottlenecks. Nevertheless many putative biomarkers have been reported from breath analysis studies even though many of these biomarkers have failed to yield reproducible results by studies made elsewhere [250,251]. A look into the breath analysis literature reveals a set of challenges and bottlenecks and they are as follows: (a) the data produced in breath analysis are biologically complex and are very large in size; (b) the data contain several sources of variance, which include information of interest, but also irrelevant variance associated with biological variation or noise [252]; (c) reported studies have very often used insufficient number of patient and control samples as compared to the large number of VOCs measured [251] which results in false positive correlations (the voodoo correlations, reviewed in [253]) and (d) many of the studies suffered from confounding variables and statistical misconceptions [251,253]. Hence, the choice of an appropriate statistical method, or data visualization tools, for detection of significant trends, correlations and, eventually, biomarkers is an important step in data mining [178]. Nevertheless, before the true statistical analysis, several important steps must be followed in order to improve the reliability of the results.

### 4.1. Data Pre-Processing and Normalization

Data pre-processing is the initial treatment of the raw data obtained after sample analysis and include several sequential paths: denoising and baseline correction, samples alignment, peak picking and merging and data matrix assembly [252]. Denoising, also known as *smoothing*, is the reduction of the noise introduced by random variations due to instrumental conditions. Similarly, the baseline correction is the correction of the background of the chromatogram. The next step is the alignment of all samples, which is necessary to reduce the distortions in retention time produced by variations in instrumental conditions (e.g., variation in temperature, column). The peak picking and merging data matrix assembly is necessary to obtain greater reliability in the results [252]. This is performed by finding all local maxima and associated local minima for each peak, as well as S/N determination. Therefore, peaks are only considered if they have S/N above a given threshold (taking into account standard deviation of the signal). Following this, an automated peak matching, based on the spectral signature, enables the representation of each VOC as a single number in the data matrix across all measured samples. The resulting data can be therefore represented as matrix whose rows are the observations and columns the relative VOCs amounts [252]. Data normalization is, particularly, an important step to simplify the statistical analysis. Typically, a normalization factor, as total area, is applied to all samples in the assumption that the total area is constant between samples, and the total profile is directly proportional to the total concentration of the sample. Other methods can be used in order to normalize the results, such as the total sum normalization, which uses the inverse of the total area aggregating normalization, known as “histogram” normalization. A detailed review about this issue can be found elsewhere [252]. As already mentioned breath analysis involves huge number of VOCs accompanied with an inherent noise. It is therefore pertinent choose a limited number of VOCs before they are developed as biomarkers. As a starting point, this selection should involve the VOCs that show highest variance or those that are mostly associated with patient sample set [179].

### 4.2. Data Analysis

Traditionally, researchers used *p*-values as a guide to identify VOCs that show statistically significant variation between the patient and control sample. However, *p*-value usage is very limited and if determined using a wrong statistical treatment may lead to false positives and negatives. Particularly, the *p*-value < 0.05 that is very often used, is not the most appropriate threshold for studies involving multiple hypothesis testing, leading to the detection of false positives [251]. Therefore, a more stringent threshold value with Bonferroni correction has been recommended [251]. Nevertheless, multivariate statistical analysis seems much more suitable to find a set of biomarkers for many different diseases and this assumption is nowadays widely consensual [252,253,254]. This can be done in an unsupervised and supervised approach. Normally, unsupervised analysis is the first approach applied in the data generated after sample analysis. It allows data exploration in order to find biological changes between the study groups. The most common method used is the Principal Component Analysis (PCA), which essentially does a reduction in the multidimensional data. However, it does not provide information regarding the variable responsible for the clustering and the outliers are a major concern [252]. Another method used is the clustering, which is a data mining technique to make automatic data groupings according to their degree of similarity. There are different clustering approaches, of which *k-means* is very often used [255,256]. However to deal with many outliers and complex data sets, more robust and sophisticated approaches should be used, as distribution-based (e.g., Gaussian mixture models [252]) or graph-based clustering methods (spectral or transitivity clustering [252,257]). The unsupervised methods are popular for data visualization but very limited to develop a classification model (do not allow data classification). Therefore, unsupervised methods are usually followed by the supervised methods which allow the characterisation of the relation between a matrix of predictors (in this case VOCs) and vector (or matrix) of responses (e.g., class membership) [252]. To achieve this, linear or nonlinear statistical techniques can be applied, according to the purpose of the analysis. In the first case, the linear discriminate analysis (LDA) is the most often used in breath analysis. Its objective is discovering a linear function based on the original compounds founded, in order to distinguish between the studied groups (e.g., control and disease groups) [258]. LDA is a fast and powerful technique, requiring few optimizing operations, but it can be only applied when there are more samples than the variables [259]. This is, however, the opposite of the most frequent situation in breath analysis and thus PCA is previously used for dimensionality reduction and only then LDA can be applied for classification purpose [252]. Partial least-square (PLS) was originally developed for the quantitative analysis in regression problems [260], but its ability to cope with highly collinear data made it also very suitable for breath analysis [261]. Using this method, it is possible to distinguish the studied classes and to find which compounds are responsible for each of them [252]. PLS-DA is another important method for samples classification. It requires some model optimization, as it frequently causes data overfitting, but it is advantageous in the fact that its model precision improves as the number of relevant variables increases [262]. Metabolomics data frequently show non-linear patterns, but these problems are well handled by using non-linear methods. There are many non-linear methods available, but the most often used are the Kernel-based models (as the support vector machine (SVM) and kernel-PLS-DA [263]), and artificial neural network (ANNs) ([264,265]). SVM method is primarily a binary classification method and in simple terms it converts the data via specific functions called kernels, to obtain a big map of nonlinear variables [252]. Hence, nonlinear techniques, particularly Kernel methods, are more powerful in terms of prediction accuracy and discrimination and are therefore naturally suitable for classification of volatomics data into, for instance, cancer and non-cancer categories [266,267]. More recently, ANNs are becoming popular in medical diagnosis [264]. ANNs is a fast, robust and highly predictive approach, involving powerful and flexible methods (it is a Euclidean metric based system that can handle complex data space) to model nonlinear problems. Although the interpretation of the effects of a compound is complicated, variants of ANNs have been applied in the analysis of sensor array results, MS data, and IMS measurements, among others [252]. An independent study using chemical sensors, for instance, has shown that ANNs is a preferred algorithm over other pattern recognition methods. This comparison was based on qualitative criteria (like speed, robustness of outlier, training difficulty, memory requirement and ability to produce measure of uncertainty), as well as quantitative criteria (classification accuracy) [268].

As already described, most EB studies involve a small sample size and a large number of variables. As a consequence, the statistical models developed can often suffer from overlearning or data overfitting [251,269,270]. Hence, a proper validation step is mandatory to increase the quality and robustness of the results. Ideally, this should include both a cross-validation as well as an external validation. Accordingly, in the cross-validation the datasets are randomly divided into “n” subsets of equal size. The model is trained using (n-1) subsets and tested using the remaining subset, followed by the calculation of performance measures, such as accuracy, true and false positives, *etc.* The training and testing is repeated till all the subsets are used as test sets. The same performance measures (sensitivity, specificity and accuracy) are then calculated as average values of all the “n” tests. It is also possible to use the “leave one out cross-validation” (LOO), where “n” is equal to the data size. The following external validation should be performed with a totally new dataset obtained from unchallenged samples (also designated as blind samples) data which is equivalent to repetition of the experiments (using a similar sample size) on independent samples obtained from the originally tested population [270]. This strict and rigorous validation protocol is particularly pertinent when using data obtained using e-noses research [250]. In summary, the choice of the best data mining procedure involves most often an option between interpretation and accuracy because more robust data mining methods involve simultaneously a harder interpretation. Moreover, it is crucial build models using an analysis of large number of the samples. This, however, is often not possible, constituting a serious limitation to the discovery of robust biomarkers [270]. This is particularly true for EB VOCs suggested as diseases biomarkers. Invariably, as can be seen from Table 1, there are many EB VOCs that are described as potential biomarkers for different diseases, but they failed when applied in a typical clinical setup. To address this problem, it is desirable to have a standard protocol of guidelines for evaluating an EB analysis study. Such protocol/guidelines have been laid down as STARD guidelines for validating diagnostic tests [271]. These comprise a list of criteria to be used when evaluating a study that proposes a biomarker, before it is used for clinical application [272,273]. The main goal of this initiative is improve the analytical performance that is important for the characterisation of a disease biomarker, namely the necessary sensitivity to indicate the exclusion of a disease and specificity to distinguish between diseases. Additionally, processing of EB analysis data should be more consistent and include not only the analytical performance results (sensitivity, specificity, or provide receiver operator characteristic (ROC) curves with associated confidence intervals), as well as information about the statistical models used [270]. This is crucial for the replication of the results by other researchers and establishment of the best methodological tools for the characterisation of EB biomarkers. Another issue that would strength the utility of EB research in disease diagnose is the improvement of data fusion possibilities, developing tools to allow the merging of data obtained from different EB analysis approaches. Regarding this, Cunha *et al.* identified putative biomarkers for tuberculosis exploiting a data collection system that uses a differential mobility spectrometer (DMS) in parallel with a MS. With this approach, they could use synergistically the statistical tools previously developed for DMS in MS chemical identification [274]. Nevertheless, more research is required to allow merger of data obtained using the state of art approaches (GC-MS and PTR-MS, for instance) with the more recent POC e-noses technologies. Furthermore, integration of EB data with other biomarkers along with patients information (blood and urinary markers, medical history, existence of risk factors for a given disease, as being smoker and lung cancer, *etc.*) will perhaps pave way for a major breakthrough towards finding clinically usable non-invasive early disease diagnose.

## 5. Conclusions

Breath analysis is still in its infancy even though EB as a means to investigate VOCs was reported some forty years ago by Pauling *et al.* [18]. Nevertheless, many improvements have been made since that time, namely at the methodological point of view with the utilization of PTR-TOF-MS, as well as a plethora of sensing system and devices. The lacking of standardized sampling collection and data processing methodologies continue to constitute serious bottlenecks. Thus, a common EB analysis and data processing standard for the data generated from different platforms (GC-MS, PTR-MS, sensors, *etc.*) and storage of such data in databases as the Human Metabolome Database [275] and CanSAR [276], is a promising strategy to increase our understanding of human metabolism in health and disease and will certainly become an important trend very shortly. At another level, traditional VOCs data analysis is still quite cumbersome and time-consuming, requiring highly skilled personnel [23]. The variety of data mining resources available nowadays is also very large and difficult to understand. Therefore, further refinement of sampling techniques, exploring advanced statistical techniques on the multi-data of VOCs to build diagnostic and prognostic models and the search for new tools that combine the strengths of the eNose (cheap, time efficient), IMS (real-time), and GC-MS (sensitive and comprehensive, allowing compounds identification) would facilitate the introduction of VOCs analysis into clinical practice [23] through POC devices. In our view, breath analysis should be integrated with the current diagnostic tools to the early disease diagnosis very efficient.

**Figure 5:**
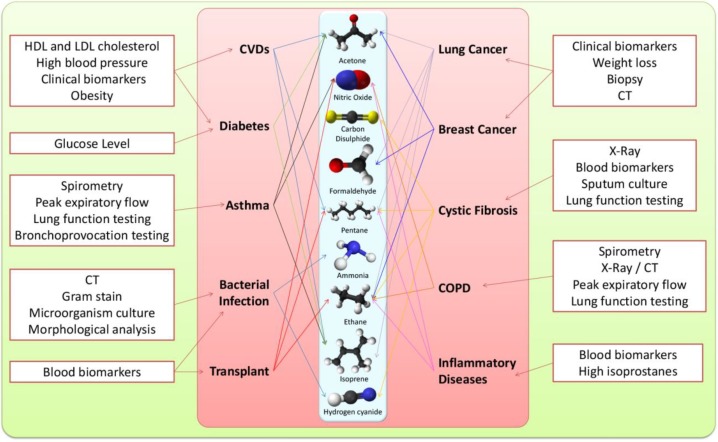
Workflow integrating breath analysis data with current diagnostic parameters to accelerate disease diagnose.

At another level, POC devices are mandatory for the use of EB analysis in the clinical environment. Unfortunately, most of the devices available are still prototypes and they are very far from coping with the complexity of EB analysis and substituting GC-MS and PTR-TOF-MS. Powerful bio-based sensor systems integrating cellular sensors and chip technology [277] and biological and chemical sensors based on graphene materials [278] are the new POC devices that would become available soon for clinical environment.

## Supplementary Files

Supplementary File 1

## Abbreviations

ACN: acetonitrile
ADH: Alcohol dehydrogenase
ADHF: Acute decompensated heart failure
AIDS: acquired immunodeficiency syndrome
AH: Alcoholic hepatitis
ALDH: aldehyde dehydrogenase
ANNs: artificial neural networks
TD: Thermal desorption
ATP: Adenosine triphosphate
AUC: area under the curves
BADD: breath analysis based disease diagnosis
CAR: Carboxen
CF: cystic fibrosis
CKD: chronic kidney disease
CO: carbon monoxide
CO_2_: carbon dioxide
COPD: Chronic Obstructive Pulmonary Disease
CRC: colorectal cancer
CT: computed tomography
CVDs: Cardiovascular diseases
DMPP: dimethylallyl pyrophosphate
DMA: Dimethyl amine
DNA: Deoxyribonucleic acid
DVB: Divinylbenzene
EB: exhaled breath
EBC: exhaled breath condensate
EDRF: endothelium-derived relaxing factor
EESI-MS: Extractive ElectroSpray Ionization Mass Spectrometry
FDH: formaldehyde dehydrogenase
FENO: NO fractional concentration in exhaled breath
FID: Flame ionization detector
HS-SPME: head-space solid phase micro extraction
FQDM: Fisher’s Quadratic Discriminant Method
GC: gas chromatography
GC-FID: gas chromatography combined with Flame ionization detector
GC-IMS: gas chromatography combined with ion mobility spectrometer
GC-MS: gas chromatography combined with mass spectrometry
GC-TOF-MS: gas chromatography combined with time-of-flight mass spectrometry
GSH: glutathione
HCA: hierarchical clustering analysis
HF: Heart Failure
HMG-CoA reductase: 3-hydroxy-3-methyl-glutaryl-CoA reductase
IBD: Inflammatory Bowel Disease
ICU: Intensive care unit
IL-4: interleukin-4
IMS: ion mobility spectrometry
iNOS: inducible NO synthase
k-NN: k-nearest neighbour
LC: Lung cancer
LDA: linear discriminate analysis
LDCT: low dose computed tomography
LOD: limit of detection
MCCV: Monte Carlo cross-validation
MDA: malondialdehyde
MEPS: microextraction by packed sorbent
MLR: Multiple Linear Regressions
MVA: multivariated analysis
MS: mass spectrometry
m/z: mass to-charge ratio
NAFLD: Non-Alcoholic Fatty Liver Disease
NDDs: neurodegenerative diseases
NIR: near infrared spectroscopy
NLST: National Lung Screening Trial
N_2_: nitrogen
NNK: 4-(methylnitrosamino)-1-(3-pyridyl)-1-butanone
NO: nitric oxide
NSCLC: Non-Small Cell Lung Carcinoma
NTD: Needle Trap Device
O_2_: oxygen
ODs: Oncologic diseases
ppm: parts per million
ppb: parts per billion
ppt: parts per trillion
PCA: Principal Component Analysis
PDMS: Polydimethylsiloxane
PFA: perfluoroalkoxy polymer
PLS: Partial least-square
PNN: Probabilistic neural network
POC: point of care
PTFE: polytetrafluoroethylene
PVDC: polyvinylidene chloride
PVF: polyvinyl fluoride
PTR: proton transfer reaction
PTR-MS: proton transfer reaction with mass spectrometry
PTR-TOF-MS: proton transfer reaction with time-of-flight mass spectrometry
ROC: Receiver operator characteristic
RNS: reactive nitrogen species
ROS: reactive oxygen species
SIFT-MS: Selected ion flow tube mass spectrometry
SIMCA: soft independent modelling of class analogy
SPME: solid phase micro extraction
SVM: support vector machine
S/N: signal-to-noise ratio
TD-tubes: thermal desorption tubes
TGF-β: transforming growth factor
TMA: trimethyl amine
TNF: tumour necrosis factor
TOF-MS: proton transfer reaction with time-of-flight mass spectrometry
VOCs: volatile organic compounds
WDA: weighted digital analysis
