# Supplementary figures and images for: Breath Analysis as a Potential and Non-Invasive Frontier in Disease Diagnosis: An Overview
Source: Metabolites. 2015 Jan 9;5(1):3–55. doi: 10.3390/metabo5010003 (PMC4381289; doi:10.3390/metabo5010003)

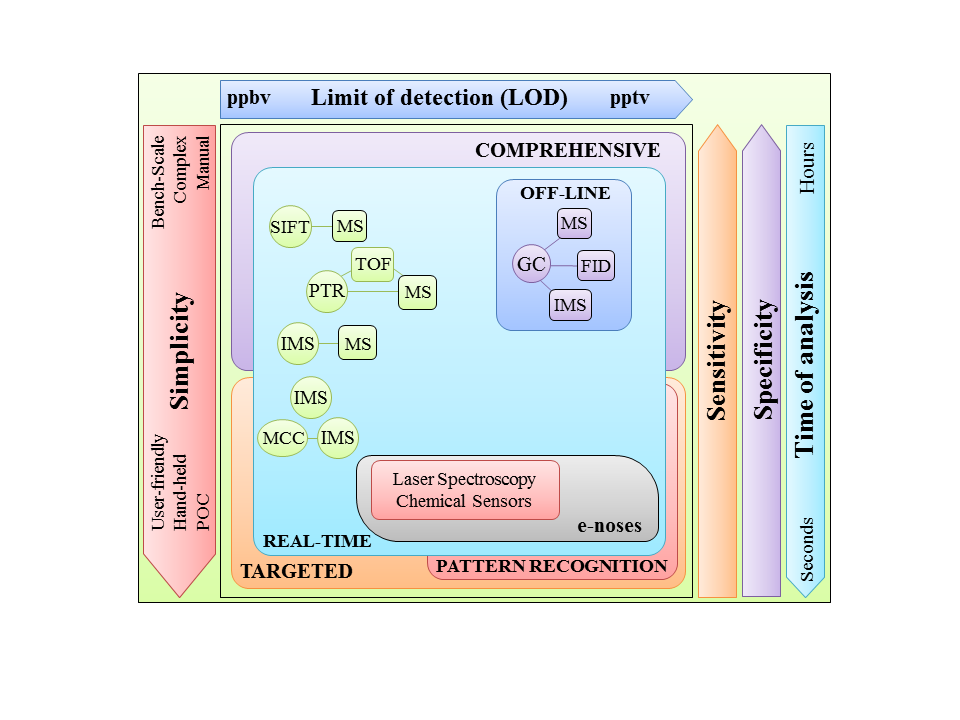

Supplement: Supplementary File 1 [file metabolites-05-00003-s001.tif]
